# EVIDENCE-BASED REVIEW & RECOMMENDATIONS FROM THE BRAZILIAN SOCIETY OF SHOULDER AND ELBOW SURGERY: REVERSE VERSUS ANATOMIC TOTAL ARTHROPLASTY FOR THE TREATMENT OF PRIMARY GLENOHUMERAL OSTEOARTHRITIS WITH AN INTACT ROTATOR CUFF IN PATIENTS AGED 70 YEARS OR OLDER

**DOI:** 10.1590/1413-785220263401e306296

**Published:** 2026-08-31

**Authors:** Marcos Rassi Fernandes, Leonardo Zanesco, Joel Murachovsky, Guilherme Grisi Mouraria, Paulo Henrique Schmidt Lara, Paulo Santoro Belangero, João Felipe de Medeiros, Eduardo Angeli Malavolta

**Affiliations:** 1 Faculdade de Medicina, Universidade Federal de Goias, Goiania, GO, Brazil.; 2 Universidade de Sao Paulo, Faculdade de Medicina, Hospital das Clinicas HCFMUSP, Sao Paulo, SP, Brazil.; 3 Faculdade de Medicina do ABC, Santo Andre, SP, Brazil.; 4 Universidade Estadual de Campinas, UNICAMP, Campinas, SP, Brazil.; 5 Escola Paulista de Medicina, UNIFESP, Sao Paulo, SP, Brazil.; 6 Universidade Federal do Rio Grande do Norte, Natal, RN, Brazil.

**Keywords:** Arthroplasty, Replacement, Shoulder, Arthroplasty, Osteoarthritis, Rotator Cuff, Elderly, Artroplastia do Ombro, Artroplastia, Osteoartrite, Manguito Rotador, Idosos

## Abstract

**Introduction:**

Anatomic total shoulder arthroplasty (ATSA) has long been indicated for the treatment of degenerative joint diseases in patients with an intact rotator cuff. However, age-related rotator cuff degeneration may negatively impact outcomes. Reverse total shoulder arthroplasty (RTSA), initially indicated for cuff tear arthropathy, has progressively expanded its indications, including elderly patients with an intact rotator cuff.

**Methods:**

A systematic review of the literature was conducted in the PubMed, Embase, Cochrane Library, and Virtual Health Library databases up to November 2025, with the aim of comparing ATSA and RTSA regarding functional outcomes, complication rates, and the need for reoperation in patients aged 70 years or older with primary shoulder osteoarthritis and an intact rotator cuff.

**Results:**

Only one systematic review met the inclusion criteria, encompassing four observational studies, with a total of 2.731 arthroplasties. RTSA showed a lower reoperation rate compared with ATSA. Functional outcomes favored ATSA, although based on limited data. No statistically significant differences were observed between the techniques regarding complication rates. The overall quality of evidence was classified as very low.

**Conclusions:**

In patients aged 70 years or older with primary glenohumeral osteoarthritis and an intact rotator cuff, RTSA is associated with a lower revision rate, whereas ATSA demonstrates superior functional outcomes, with similar complication rates between the two techniques. ***Level of evidence II; Systematic review.***

## RESUMO

**Introdução:**

A artroplastia anatômica total do ombro (AATO) é tradicionalmente indicada para osteoartrite glenoumeral com manguito rotador íntegro. Contudo, a degeneração do manguito relacionada à idade pode comprometer seus resultados. A artroplastia reversa total do ombro (ARTO), inicialmente indicada para artropatia do manguito rotador, teve suas indicações ampliadas, incluindo pacientes idosos com manguito íntegro.

**Métodos:**

Realizou-se revisão sistemática da literatura nas bases PubMed, Embase, Cochrane Library e Biblioteca Virtual de Saúde, até novembro de 2025, com o objetivo de comparar a AATO e a ARTO quanto aos desfechos funcionais, às taxas de complicações e à necessidade de reoperação em pacientes com idade igual ou superior a 70 anos, portadores de osteoartrite primária do ombro e manguito rotador íntegro.

**Resultados:**

Apenas uma revisão sistemática atendeu aos critérios de inclusão, englobando quatro estudos observacionais, com total de 2,731 artroplastias. A ARTO apresentou menor taxa de reoperação em comparação à AATO. Os desfechos funcionais favoreceram a AATO, embora baseados em dados limitados. Não foram observadas diferenças estatisticamente significativas entre as técnicas quanto à taxa de complicações. A qualidade da evidência foi classificada como muito baixa.

**Conclusões:**

Em pacientes com idade igual ou superior a 70 anos, com osteoartrite glenoumeral primária e manguito rotador íntegro, a ARTO associa-se a menor taxa de reoperação, enquanto a AATO apresenta melhores desfechos funcionais, com taxas de complicações semelhantes entre as duas técnicas. ***Nível de evidência II; Revisão sistemática.***

## RECOMMENDATION

There is very low-quality evidence that reverse total shoulder arthroplasty (RTSA) is superior to anatomic total shoulder arthroplasty (ATSA) regarding the reoperation rate in the treatment of primary glenohumeral osteoarthritis in patients with an intact rotator cuff aged 70 years or older.

With respect to functional outcomes, there is weak scientific evidence indicating that ATSA is superior to RTSA in this population. There is very low-quality evidence that both modalities present similar results regarding the complication rates in the studied population.

Therefore, the Brazilian Society of Shoulder and Elbow Surgery Consensus Project recommends that the choice between RTSA and ATSA be individualized, considering, on a case-by-case basis, patient-related factors, expectations, functional demands, physical examination findings, and imaging results, in order to guide the decision toward the most appropriate procedure.

From a practical standpoint, the findings suggest that RTSA may represent an alternative in patients at greater risk of long-term rotator cuff functional failure, whereas ATSA tends to provide better functional parameters, especially with regard to rotational mobility. Thus, the choice of prosthesis type should consider not only the average reported outcomes but also the individual patient's expectations.

## INTRODUCTION

ATSA has a long history in the treatment of degenerative joint diseases in patients with an intact rotator cuff, with several studies reporting satisfactory outcomes.^1,2^ However, in the presence of rotator cuff tears or when severe atrophy associated with fatty infiltration is present, clinical outcomes tend to be unfavorable.^3,4^

Rotator cuff tendons undergo progressive degeneration with advancing age, accompanied by secondary muscle belly atrophy, and injuries are present in nearly 50% of the population in the eighth or ninth decades of life.^5–7^ Furthermore, even in the absence of tendon rupture, fatty degeneration of the rotator cuff muscles has been associated with worse clinical outcomes, reduced range of motion, and lower satisfaction after ATSA.^4^

On the other hand, RTSA was initially introduced as a treatment for rotator cuff arthropathy. Over the years, its indications have progressively expanded, with an exponential increase in frequency of use. In this context, RTSA has also been indicated for the treatment of shoulder osteoarthritis, even in the presence of an intact rotator cuff, with the theoretical advantage of compensating for potential cuff dysfunction or a possible late rupture following ATSA.

However, due to its particular biomechanics, recovery of range of motion, especially medial and lateral rotations, after RTSA is less predictable when compared with ATSA. In addition, there is a potential increase in the risk of complications, such as infection and nerve injury, as well as a scarcity of long-term survivorship data.^8,9^

Given this scenario, comparative evaluations between ATSA and RTSA regarding the need for reoperation, functional outcomes, and complication rates in patients aged 70 years or older with primary shoulder osteoarthritis and an intact rotator cuff become relevant.

### Research Question

In patients aged 70 years or older with primary glenohumeral osteoarthritis and an intact rotator cuff, which surgical modality is more effective, ATSA or RTSA, considering the need for reoperation, functional outcomes, and complication rates?

## METHODS

- Databases searched: PubMed, Embase, Cochrane Library, and Virtual Health Library (VHL).
- Descriptors: "Shoulder Joint"[MeSH]; "Arthroplasty, Replacement"[MeSH]; "Arthroplasty, Replacement, Shoulder"[MeSH]; "Treatment Outcome"[MeSH]; "Reverse Shoulder Arthroplasty"; "Systematic Review"[Publication Type]; "Randomized Controlled Trial"[Publication Type] (see Appendix 1).
- Search period: up to November 2025.
- Study selection: updated systematic reviews (SRs) with adequate methodological quality were prioritized. In the absence of SRs, randomized clinical trials (RCTs) were considered, provided that methodological quality had been assessed.

## RESULTS

The search identified 426 references (144 in PubMed, 92 in the Cochrane Library, 144 in Embase, and 46 in the Regional Portal of the VHL). Of these, eight were SRs, the study type prioritized in this evaluation, and no RCTs were identified.

After full-text analysis, seven SRs were excluded.^10–16^ Two Cochrane reviews addressed arthroplasty in general terms, without direct comparison relevant to the clinical question.^10,11^ One was excluded for being a narrative review,^12^ and another for addressing cost-effectiveness, an outcome not intended in this analysis.^13^ Of the remaining three reviews, one included samples with other indications for arthroplasty, such as fractures and rotator cuff arthropathy,^14^ and two included patients younger than 70 years.^15,16^

**Figure 1:**
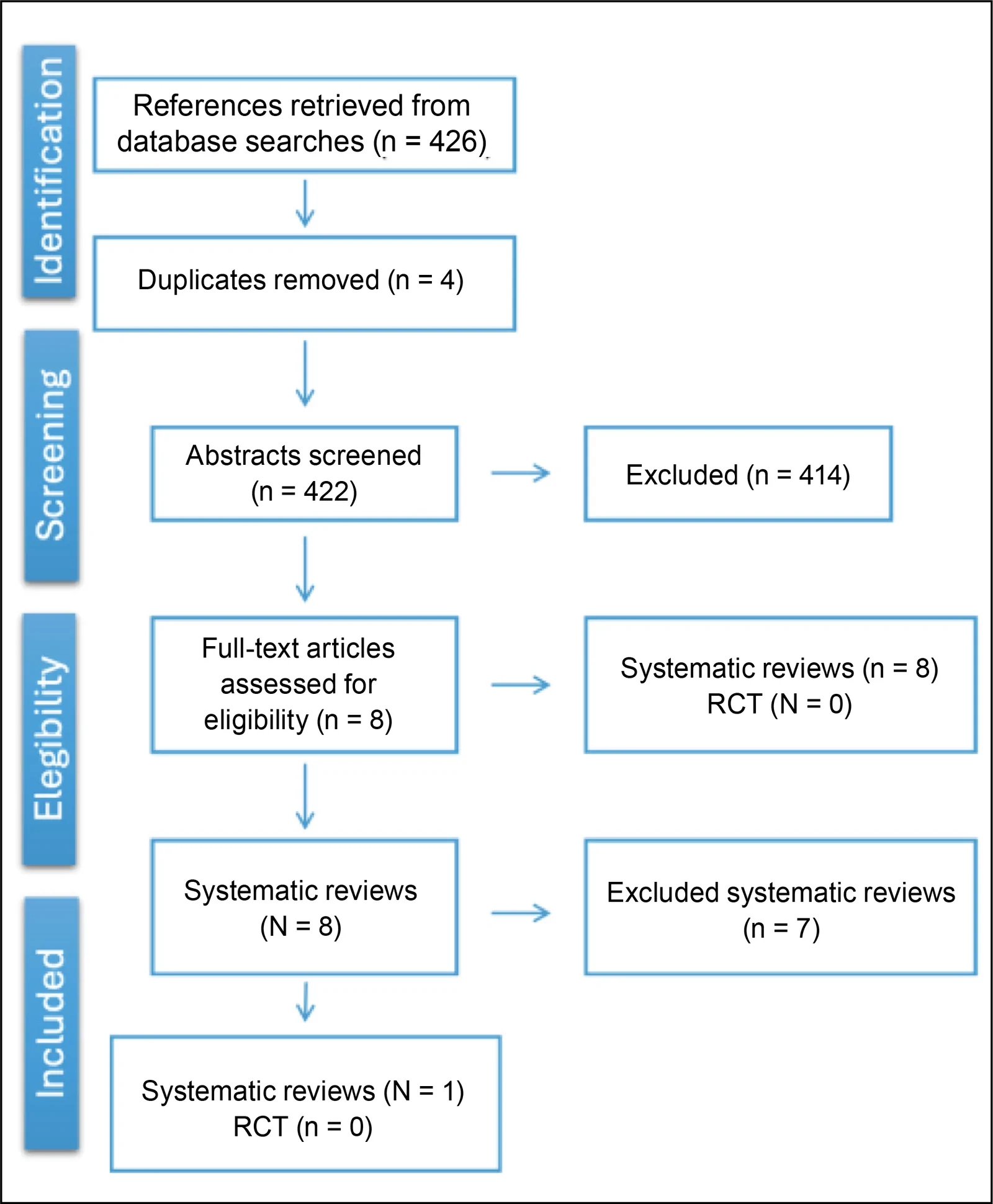
Flowchart of the literature search.

Thus, only one SR remained eligible,^17^ and no RCTs directly addressing the clinical question were identified (Figure 1).

### Included Studies

No RCTs on the topic were found. A single systematic review compared ATSA and RTSA in the treatment of primary shoulder osteoarthritis in patients with an intact rotator cuff aged 70 years or older, with a minimum follow-up of two years.^17^ The primary outcome evaluated was the surgical revision rate, while functional scores and complication rates were considered secondary outcomes.

Four studies were included in this SR, totaling 2731 arthroplasties (1472 ATSA and 1259 RTSA).^18–21^ Of these, three were retrospective cohort studies and one consisted of a registry analysis. Methodological quality was assessed using the Methodological Index for Non-Randomized Studies (MINORS), with a mean score of 18 points, ranging from 17 to 20, out of a total of 24.

All four studies compared the overall reoperation rate between ATSA and RTSA. The surgical revision rate was 3.05% for ATSA and 1.18% for RTSA (OR 0.50; 95% CI: 0.30–0.84; p < 0.05; I² = 0%).

Direct comparison of functional outcomes was not possible in this SR due to the absence of complete statistical data in the included studies, particularly the lack of standard deviation information, which precluded meta-analysis. The only available parametric analysis referred to the Constant-Murley score, derived from a single study, with results favoring ATSA (80 [75–82]) compared with RTSA (68 [66–76.5]) (p < 0.0001).^18^

Two studies provided complete data regarding types of complications and overall complications rates.^18,21^ Reported complications included periprosthetic fracture, glenoid component loosening, scapular notching, instability, deep infection, neurological injury, vascular injury, secondary rotator cuff rupture, deep vein thrombosis, and hematoma. The complication rate was 7.9% for ATSA and 8.8% for RTSA, with no statistically significant difference between groups (OR 0.98; 95% CI: 0.34–2.86; p = 0.97; I² = 0%).

The general characteristics of the eligible study and the assessment of evidence quality according to the Grading of Recommendations Assessment, Development, and Evaluation (GRADE) system^22,23^ are presented in Tables 1 and 2, respectively.

**Table 1:** Characteristics of the included Systematic Review.

	Study design	Included studies	Sample	Prior protocol publication
Dragonas et al. 2023	SR	4 Retrospective studies (18-21)	2.731	Yes (PROSPERO)

SR- systematic review.

**Table 2:** Assessment of the quality of study evidence according to the GRADE system.

Study	Outcome	Relative Effect (1C95%)	Recommendation grade
Dragonas et al. 2023	Reoperation rate	OR.5 (CI 0.30 - 0.84)	Very low
Dragonas et al. 2023	Functional outcomes	ATSA: 80 (75-82) RTSA: 68 (66- 76.5)	Very low
Dragonas et al. 2023	Complications rates	OR= 0.98 (CI 0.34 - 2.86)	Very low

Note: The GRADE system (Grading of Recommendations Assessment, Development and Evaluation) is a tool used to assess and measure the quality of evidence. It is based on five domains: risk of bias, imprecision, inconsistency, indirectness of outcomes, and publication bias. Based on these criteria, the quality of evidence is classified as very low, low, moderate, or high. OR- Odds Ratio. CI — Confidence Interval.

## CONCLUSIONS

In the comparison between ATSA and RTSA in patients aged 70 years or older with primary shoulder osteoarthritis and an intact rotator cuff, there is very low-quality evidence that RTSA is superior to ATSA regarding the reoperation rate.

Regarding functional outcomes, there is very low-quality evidence indicating superiority of ATSA over RTSA. There is also very low-quality evidence that both techniques present similar results regarding complication rates.

### Implications for Research

Future research should prioritize the conduct of randomized, multicenter clinical trials with rigorous methodology. Studies with this design are essential to more precisely define long-term clinical and functional outcomes associated with each surgical modality, since no studies with high methodological rigor on this topic were identified.

## Data Availability

The data underlying this study are available within the article.

## Appendix 1 Search Strategies Across Databases.

**Table:**

**PUBMED**
#1"Shoulder Joint"[Mesh] OR (Joint, Shoulder) OR (Joints, Shoulder) OR (Shoulder Joints) OR (Glenohumeral Joint) OR (Glenohumeral Joints) OR (Joint, Glenohumeral) OR (Joints, Glenohumeral) OR (GlenoidLabrum) OR (Labrum, Glenoid) OR (Glenohumeral Joint) OR (Shoulder Arthritis) OR (Glenohumeral Arthritis) OR (Shoulder Osteoarthritis)
#2 "Arthroplasty, Replacement"[Mesh] OR (Joint Replacement) OR (Joint Replacements) OR (Replacement, Joint) OR (Replacements, Joint) OR (Arthroplasties, Replacement) OR (Replacement Arthroplasties) OR (Replacement Arthroplasty) OR (Joint Prosthesis Implantation) OR (Implantation, Joint Prosthesis) OR (Implantations, Joint Prosthesis) OR (Joint Prosthesis Implantations) OR (Prosthesis Implantation, Joint) OR (Prosthesis Implantations, Joint) OR (Total Joint Replacement) OR (Joint Replacements, Total) OR (Joint Replacement, Total) OR (Replacements, Total Joint) OR (Replacement, Total Joint) OR (Total Joint Replacements) OR (Arthroplasty, Replacement, Reverse Shoulder) OR (Reverse Shoulder Arthroplasty)
#3 "Arthroplasty, Replacement, Shoulder"[Mesh] OR (Shoulder Replacement Arthroplasty) OR (Arthroplasties, Shoulder Replacement) OR (Arthroplasty, Shoulder Replacement) OR (Replacement Arthroplasties, Shoulder) OR (Replacement Arthroplasty, Shoulder) OR (Shoulder Replacement Arthroplasties) OR (Total Shoulder Replacement) OR (Replacements, Total Shoulder) OR (Replacement, Total Shoulder) OR (Shoulder Replacements, Total) OR (Shoulder Replacement, Total) OR (Total Shoulder Replacements) OR (Arthroplasty, Replacement, Shoulder) OR (Anatomic Shoulder Arthroplasty) OR (Total Shoulder Arthroplasty)
#4 ("Treatment Outcome"[MeSH] OR "Postoperative Complications"[MeSH] OR "Reoperation"[MeSH] OR "Functional Outcome")
Filters applied: Clinical Trial, Controlled Clinical Trial, Meta-Analysis, Randomized Controlled Trial, Systematic Review, Aged: 65+ years, 80 and over: 80+ years.
#5 #1 AND #2 AND #3 AND #4

**Table:**

**COCHRANE LIBRARY**
#1 (Joint Shoulder) OR (JointsShoulder) OR (Shoulder Joints) OR (Glenohumeral Joint) OR (Glenohumeral Joints) OR (Joint Glenohumeral) OR (Joints, Glenohumeral) OR (GlenoidLabrum) OR (LabrumGlenoid) OR (Glenohumeral Joint) OR (Shoulder Arthritis) OR (Glenohumeral Arthritis) OR (Shoulder Osteoarthritis)
#2 (Arthroplasty Replacement) OR (Joint Replacement) OR (Joint Replacements) OR (Replacement Joint) OR (Replacements Joint) OR (Arthroplasties Replacement) OR (Replacement Arthroplasties) OR (Replacement Arthroplasty) OR (Joint Prosthesis Implantation) OR (Implantation Joint Prosthesis) OR (Implantations Joint Prosthesis) OR (Joint Prosthesis Implantations) OR (Prosthesis Implantation Joint) OR (Prosthesis Implantations Joint) OR (Total Joint Replacement) OR (Joint Replacements Total) OR (Joint Replacement Total) OR (Replacements Total Joint) OR (Replacement Total Joint) OR (Total Joint Replacements) OR (Arthroplasty Replacement Reverse Shoulder) OR (Reverse Shoulder Arthroplasty)
#3 (Arthroplasty Replacement Shoulder) OR (Shoulder Replacement Arthroplasty) OR (Arthroplasties Shoulder Replacement) OR (Arthroplasty Shoulder Replacement) OR (Replacement Arthroplasties Shoulder) OR (Replacement Arthroplasty Shoulder) OR (Shoulder Replacement Arthroplasties) OR (Total Shoulder Replacement) OR (Replacements Total Shoulder) OR (Replacement Total Shoulder) OR (Shoulder Replacements Total) OR (Shoulder Replacement Total) OR (Total Shoulder Replacements) OR (Arthroplasty Replacement Shoulder) OR (Anatomic Shoulder Arthroplasty) OR (Total Shoulder Arthroplasty)
#4 (Treatment Outcome) OR (Postoperative Complications) OR Reoperation OR (Functional Outcome)
#5 (Aged, 80 and over) OR (OlderAdults) OR (Elderly)
#6 #1 AND #2 AND #3 AND #4 AND #5

**Table:**

**EMBASE**
#1 ‘shoulder joint’/exp OR ‘articulatio glenohumeralis’ OR ‘articulatio humeri’ OR ‘gleno-humeral joint’ OR ‘glenohumeral joint’ OR ‘humeral joint’ OR ‘humero-scapular joint’ OR ‘humeroscapular joint’ OR ‘joint, glenohumeral’ OR ‘joint, humeroscapular’ OR ‘scapulohumeral joint’ OR ‘scapulohumeral joint’ OR ‘shoulder (joint)’ OR ‘shoulder joint’OR (GlenoidLabrum) OR (Labrum, Glenoid) OR (Glenohumeral Joint) OR (Shoulder Arthritis) OR (Glenohumeral Arthritis) OR (Shoulder Osteoarthritis)
#2 ‘Replacement Arthroplasty’/exp OR ‘arthroplasty, replacement’ OR ‘joint replacement’ OR ‘joint replacementsurgery’ OR ‘Replacement Arthroplasty’ OR ‘replacement, joint’ OR (Implantation Joint Prosthesis) OR (Implantations Joint Prosthesis) OR (Joint Prosthesis Implantations) OR (Prosthesis Implantation Joint) OR (Prosthesis Implantations Joint) OR (Total Joint Replacement) OR (Joint Replacements Total) OR (Joint Replacement Total) OR (Replacements Total Joint) OR (Replacement Total Joint) OR (Total Joint Replacements) OR (Arthroplasty Replacement Reverse Shoulder) OR (Reverse Shoulder Arthroplasty)
#3 (Arthroplasty Replacement Shoulder) OR (Shoulder Replacement Arthroplasty) OR (Arthroplasties Shoulder Replacement) OR (Arthroplasty Shoulder Replacement) OR (Replacement Arthroplasties Shoulder) OR (Replacement Arthroplasty Shoulder) OR (Shoulder Replacement Arthroplasties) OR (Total Shoulder Replacement) OR (Replacements Total Shoulder) OR (Replacement Total Shoulder) OR (Shoulder Replacements Total) OR (Shoulder Replacement Total) OR (Total Shoulder Replacements) OR (Arthroplasty Replacement Shoulder) OR (Anatomic Shoulder Arthroplasty) OR (Total Shoulder Arthroplasty)
#4 (Treatment Outcome) OR (Postoperative Complications) OR Reoperation OR (Functional Outcome)
#1 AND #2 AND #3 AND #4 AND #5 AND ([aged]/lim OR [veryelderly]/lim) AND ([cochranereview]/lim OR [systematicreview]/lim OR [meta analysis]/lim OR [controlled clinical trial]/lim OR [Randomized Controlled Trial]/lim)

**Table:**

**REGIONAL PORTAL VHL**
#1 MH:"Articulação do Ombro" OR (Shoulder Joint) OR (Articulação Glenoumeral) OR (Articulações dos Ombros) OR (Ligamento Glenoide Umeral) OR (Lábio Glenoidal) OR MH:A02.835.583.748$ OR (Articulação do Ombro) OR (Artrite Glenoumeral) OR (Articulação do Ombro) OR (Osteoartrite do Ombro) OR (Artrose Glenoumeral)
#2 MH:"Artroplastia do Ombro" OR (Arthroplasty, Replacement, Shoulder) OR (Substituição Total do Ombro) OR MH:E04.555.110.110.299$ OR MH:E04.617.101.110.299$ OR MH:E04.650.110.299$ OR (Artroplastia Reversa do Ombro) OR (Prótese Reversa do Ombro)
#3 (Artroplastia Anatômica do Ombro) OR (Artroplastia Total do Ombro)
#4 (Idoso) OR (Idoso de 80 anos ou mais)
#5 #1 AND #2 AND #3 AND #4
